# Relationship satisfaction and family routines of young parents before and during the first year of the COVID-19 pandemic: A latent growth curve analysis

**DOI:** 10.1371/journal.pone.0297740

**Published:** 2024-02-16

**Authors:** Victoria Weise, Felicitas Güttner, Andreas Staudt, Judith T. Mack, Susan Garthus-Niegel

**Affiliations:** 1 Institute and Outpatient Clinics of Occupational and Social Medicine, Faculty of Medicine, Technische Universität Dresden, Dresden, Germany; 2 Department of Methods in Community Medicine, Institute for Community Medicine, University Medicine Greifswald, Greifswald, Germany; 3 Institute for Systems Medicine and Faculty of Medicine, Medical School Hamburg, Hamburg, Germany; 4 Department of Childhood and Families, Norwegian Institute of Public Health, Oslo, Norway; University of Wrocław: Uniwersytet Wroclawski, POLAND

## Abstract

With the onset of the COVID-19 pandemic, parents had to reorganize their family routines under many external stressors (e.g., limited external childcare), which could have negatively affected their relationship satisfaction. This study aimed to examine the changes in relationship satisfaction of young parents from pre-pandemic times up to the second wave of the COVID-19 pandemic in Germany in December 2020 and whether these changes were different for mothers and fathers. Additionally, the role of perceived pandemic-related stress and changes in family routines was investigated. Data from 564 participants from DREAM_CORONA_, a sub-study of the prospective longitudinal cohort study “Dresden Study on Parenting, Work, and Mental Health” (DREAM), were analyzed. Relationship satisfaction was assessed at three measurement points (T0: pre-pandemic, i.e., August 2018–March 2020; T1: May–June 2020; T2: October–December 2020). To estimate changes in relationship satisfaction over time, Latent Growth Curve Models were calculated. Changes in family routines (i.e., changes in the division of housework and childcare from T0 to T1 as well as the availability of external childcare facilities at T1) and perceived pandemic-related stress at T1 were used as predictors. The models were adjusted for education and number of children per household. There was no significant change in relationship satisfaction over time, with no differences between mothers and fathers. The multi-group model revealed that changes in the division of housework and childcare predicted changes in relationship satisfaction in mothers, but not in fathers. For mothers, doing more housework than before the pandemic was negatively associated with changes in their relationship satisfaction over time. Additionally, reporting that their partner did more childcare than before the pandemic was positively associated with the relationship satisfaction of mothers. Our results indicate no general negative prospective association between the COVID-19 pandemic and parental relationship satisfaction over time. Nevertheless, our findings highlight the importance of the division of housework and childcare for mothers’ relationship satisfaction and how pandemic-related changes in family routines alter this association.

## Introduction

### Being a couple throughout a worldwide pandemic

The COVID-19 pandemic changed daily life in nearly every country worldwide. To stop the spreading of *SARS*-*CoV*-*2*, governments all over the world had to set new regulations, including social distancing, lockdowns, working from home, closure of childcare facilities, and more. As known from evidence from outside the context of the COVID-19 pandemic, both major stress and life events (e.g., natural disasters) and minor stress (e.g., work stress) from external sources can have a negative spillover on the couple relationship [1–3]. Applying the stress transactional model [STM; 2, 4], stress from outside the partnership, i.e., extradyadic stress, can increase intradyadic stress and impair mental well-being and relationship satisfaction [5, 6]. In the context of the COVID-19 pandemic in particular, both extradyadic stress (e.g., related to pandemic-related restrictions or increase in anxiety and worry) [7–9] and intradyadic stress (e.g., more conflicts and dysfunctional dyadic processes) [1, 10] were prone to negatively affect relationship satisfaction.

However, studies that investigated changes in couples’ relationship satisfaction during the COVID-19 pandemic yielded inconsistent findings [for an overview see 11]. Some studies did not detect any [12] or just a minimal decrease [13] in overall relationship satisfaction among cohabiting couples. For instance, in a cross-national study with 1,125 participants from 57 countries, increased stress at the onset of the pandemic was negatively associated with relationship satisfaction cross-sectionally and over the following three months [14]. Furthermore, longitudinal data from Germany showed a steeper decrease in relationship satisfaction during the first wave of the COVID-19 pandemic in the early summer of 2020 compared to data from the years 2018 and 2019 [15]. Several other studies also reported a negative role of the COVID-19 pandemic for relational well-being. One study found a general increase in relationship turbulence caused by the COVID-19 pandemic [16] and two studies with American populations reported that 20–34% of their participants experienced conflicts in their relationships due to the COVID-19 pandemic [9, 17]. What is more, a recent meta-analysis found a decrease in sexual behaviors and satisfaction during the COVID-19 pandemic [18]. However, there are some studies reporting an increase in relationship satisfaction during the pandemic [19, 20]. Possible reasons might be that some couples might have benefitted from managing the pandemic-related stress together, due to an increase in dyadic coping [21, 22], which is positively associated with relational well-being and relationship satisfaction [5, 23]. Still, one study found that "overall couple members’ experiences were quite polarized” [24, p. 2246], i.e., half of their participants were “happy to be home” during the pandemic and reported better relationships satisfaction, whereas for others being at home was unpleasant and strained the relationship. The authors discuss that these groups may differ in terms of multiple stressors and presence of children, with couples being “happy to be home” having more resources to resist adverse conditions during the pandemic. Taken together, the prevailing literature indicates that the COVID-19 pandemic was a potential burden on relationship satisfaction, although it remains an open question whether all couples were equally affected. In this context, the underlying dynamics might be more complex than expected.

Sex differences in changes in relationship satisfaction also need to be investigated further. Meta-analytic evidence from before the COVID-19 pandemic showed that relationship satisfaction changes over a lifetime; however, no sex differences were detected in community samples across studies [25, 26]. In general, couples with children experience lower relationship satisfaction than childless couples [25, 27]. Interestingly, it was found that this difference was largest for mothers with infants, as evidenced by 38% of mothers compared to 62% of childless women reporting high relationship satisfaction; in contrast, the effect was similar for all ages of their children among fathers [27]. Further, the number of children was found to be negatively associated with relationship satisfaction [27, 28].

In general, women were found to be more prone to experiencing heightened stress compared to men [e.g., 29]. In correspondence with this, a Swiss study found that even though women and men did not differ in their relationship satisfaction, women reported higher levels of extradyadic and intradyadic stress [5]. Therefore, this raises the question whether women experienced a greater decline in relationship satisfaction during the exceptionally stressful COVID-19 pandemic. Some pandemic-related studies did not observe any overall differences between women and men in (changes in) relationship satisfaction [12, 20]. On the other hand, another study found some sex differences regarding specific indicators of relationship stress [30]. For example, increased stress due to relationship conflicts was reported only by women. In this regard, it should be noted that women appeared to be more emotionally affected by the COVID-19 pandemic overall by reporting more psychological distress [31] and experiencing higher levels of stress during quarantine [32] than men. These results suggest that women and men possibly differed in their changes in relationship satisfaction due to the COVID-19 pandemic, with women potentially experiencing a greater decline.

### The special situation of parents during the COVID-19 pandemic

Parents, especially, were exposed to increased stress due to the constantly changing restrictions and the need to reorganize family routines and roles [33, 34]. In particular, parents had to cope with repeatedly changing childcare arrangements [35]. During the first lockdown in spring 2020, up to 93% of German parents had to look after their children at home because external childcare facilities were closed [36]. The additional childcare responsibilities had to be reconciled with work, which was a major source of stress or worse mental health for parents throughout the pandemic [9, 34, 37, 38] and appeared to have affected parental well-being [39] and parent-child-bonding [40, 41]. A decline in parental well-being might have in turn adversely affected parents’ relationship satisfaction [42, 43]. In parallel with childcare responsibilities, the amount of housework increased during the pandemic [9], raising the question of how parents shared the additional workload related to both domains.

Even before the pandemic, restrictive gender norms and gender inequality were deeply rooted and widespread in the global world [44]. The gender care gap is defined as the percentage difference in the average daily time women spend on unpaid work compared to men [45]. In Germany, where the present study took place and which ranks in the middle in a global comparison [46, 47], a representative survey from 2010–2020 revealed an inflationary increase in the gender care gap between the ages of 25 and 35. In particular, at the age of 34, women spent around nine hours and men spent approximately three hours per day on unpaid care work, yielding a gender care gap of 170% [48]. This coincides with the transition to (first-time) parenthood, which has been identified–even in pre-pandemic studies–as a time when gender role attitudes become more traditional and the unequal division of housework between the sexes accelerates [49–52].

In terms of possible changes in the division of unpaid work related to the additional workload resulting from the COVID-19 pandemic, we find mixed evidence. Some studies report that there was an equal increase in housework and childcare responsibilities for women and men, indicating no change to the existing gender gap, with mothers doing generally more housework and childcare than fathers [53–55]. Others point toward an increasing inequality in the division of housework and childcare, reporting that women took over most of the additional tasks [34, 56, 57]. In contrast, a study conducted on an Australian sample at the beginning of the pandemic found that, although both mothers and fathers spent more time on childcare *and* housework, fathers increased their involvement in childcare more than mothers, reducing the gender gap in childcare but not in housework [58]. However, data from Germany indicate that such a trend towards a more equal distribution of childcare declined as the pandemic progressed [59].

The way parents divide housework and childcare between themselves may affect their relationship satisfaction. To observe potential differential relations, it is recommended to consider housework and childcare separately [60, 61]. The “traditional” division of housework, with women doing most of the chores, typically results in lower relationship satisfaction for women compared to a more egalitarian division [62, 63]. In line with this, women who reported increasing household demands due to the COVID-19 pandemic showed more relationship problems and lower relationship satisfaction [64]. From the men’s perspective, there was no clear tendency for relationship quality to decrease when they were more involved in housework [62, 65]. In terms of childcare, doing most of the childcare was associated with lower relationship satisfaction in women [66, 67]. In contrast, greater involvement of fathers in childcare was associated with better relationship satisfaction ratings for both parents [67, 68]. As parents were particularly stressed by increased household and childcare responsibilities and lack of external support [32, 69], parental relationships might have suffered during the COVID-19 pandemic. Evidence from a mixed-methods study supports this hypothesis by showing high levels of dyadic adjustment in childless couples, but lower levels in parents during the lockdown in spring 2020 [19]. Further, parents reported a more conflictual atmosphere and unbalanced needs when asked about changes in their couple or family dynamics due to the pandemic. In another study, mothers and fathers showed more global distress and more difficulties with problem-solving communication compared to childless couples during the first rigorous lockdown in Belgium [70]. It should be mentioned that there are also contrary results indicating that parental relationships benefited during the pandemic [71] and that the presence of children might have buffered the pandemic-related decrease in relationship quality [15]. Nevertheless, there is substantial evidence that at least some parents experienced a decline in their relational well-being due to the COVID-19 pandemic. This is crucial because a decrease in relationship satisfaction may increase the risk of psychological distress [72] and depressive symptoms [73, 74] in parents or could even impair children’s development [75, 76]. Therefore, it is important to further investigate the changes in young parents’ relationship satisfaction during the COVID-19 pandemic and how changes in family routines and perceived pandemic-related stress could have influenced these changes. Hereby, it is important to consider the differences between mothers and fathers in their relationship satisfaction, because–as mentioned earlier–recent studies found that women experience a greater decline during the transition to parenthood [27] and are more prone to experiencing stress than men [e.g., 5, 29]. Likewise, differential maternal versus paternal changes in relationship satisfaction due to the COVID-19 pandemic are conceivable, with mothers possibly experiencing a greater decline than fathers.

### The present study

The main research question of this study, based on a preregistered analysis plan, was whether young parents’ relationship satisfaction changed from pre-pandemic times up to the first year of the COVID-19 pandemic. As part of a secondary research question, we aimed to examine the role of changes in family routines (i.e., the division of housework and childcare and the availability of external childcare facilities) and perceived pandemic-related stress at the early stages of the pandemic on changes in young parents’ relationship satisfaction over time. Given the major disruption to family life caused by the onset of the COVID-19 pandemic, we chose this prospective study design because we were particularly interested in how role division in the early days of the pandemic might be prospectively associated with changes in relationship satisfaction over time. We adjusted for number of children living in the household [77] and education [66, 78], as these factors are likely to influence relationship satisfaction. In addition, to account for the fact that participants were at different stages of the overall cohort study, we included the age of the index child (i.e., the child with whom the parents participated in the longitudinal study, which was the first child for most participating couples) as a covariate (see Study Design in the Methods section for detailed information).

Based on the evidence mentioned above, the following hypotheses were formulated:

(1) Parental relationship satisfaction decreased from pre-pandemic times up to the second wave of the COVID-19 pandemic in Germany in December 2020.

(1a) There is a difference in the extent of the decline in relationship satisfaction between mothers and fathers, with mothers experiencing a greater decrease than fathers.

The following secondary research questions investigated exploratively:

(2) Are changes in family routines (i.e., changes in the division of housework and childcare and the availability of external childcare facilities) and/or perceived pandemic-related stress during the early pandemic prospectively associated with changes in young parents’ relationship satisfaction up to the second wave of the COVID-19 pandemic in Germany, adjusting for education, number of children per household, and age of the index child?

(2a) Do these prospective associations differ between mothers and fathers?

## Methods

### Study design

Data for this study were derived from the “Dresden Study on Parenting, Work, and Mental Health” (**DREAM**; **DR**esdner Studie zu **E**lternschaft, **A**rbeit und **M**entaler Gesundheit) as well as the associated sub-study DREAM_CORONA_. The DREAM study is a prospective multi-method cohort study that aims to investigate the relations between parental work participation, role distribution, and stress factors and their impact on family health-related issues. Participants were recruited during pregnancy from June 2017 to the end of 2020, mainly at obstetrical clinics or midwife practices during birth information evenings. The inclusion criteria for expectant mothers and fathers comprised being a resident in or around Dresden, Germany, and having sufficient German skills. To take part, the participants had to complete paper or online questionnaires on somatic and mental health, family and relationship factors, as well as work-related subjects. Currently, the DREAM study has one prepartum and five postpartum measurement points until 4.5 years after childbirth [for more details, see 79]. The DREAM_CORONA_ sub-study investigates the experiences of (expectant) parents during the COVID-19 pandemic (e.g., isolation, school and daycare closures, working from home) and its impact on family health, role distributions, and relationships. Participants of the main DREAM study were invited to complete an online survey at two different time points. For feasibility reasons, participants who wished to complete follow-up questionnaires via paper version and those with multiple pregnancies had to be excluded. The first survey (T1) was sent out via e-mail on May 12, 2020, followed by two reminders after three and six days, respectively. The second survey (T2) followed in October 2020 and was sent out according to the same principle.

**Fig 1:**
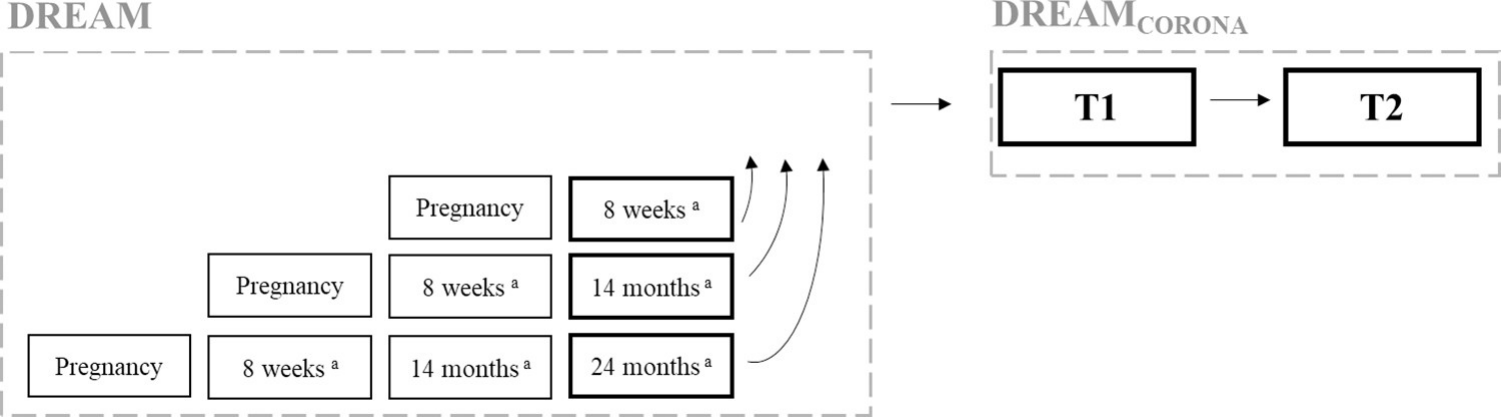
Used surveys of the main DREAM study (T0) and the DREAM_CORONA_ sub-study (T1, T2). The surveys relevant for the dependent variable relationship satisfaction are marked in bold. ^a^Time postpartum.

In this study, relationship satisfaction was analyzed across three different time points (see Fig 1) based on a preregistered analysis plan (https://osf.io/cezf4) with only small deviations that are acknowledged in the relevant sections. For the first time point (hereafter referred to as T0), we used data from the main DREAM study. For each participant, the last completed questionnaire before participation in the DREAM_CORONA_ study was used as T0 (data collection ranging from August 10, 2018 to March 5, 2020). This questionnaire could be the main DREAM survey completed at 8 weeks, 14 months, or 24 months postpartum [see study protocol; 79]. The survey of the second time point (hereafter referred to as T1) was open for participants to complete between May and August 2020 as part of the first survey of the DREAM_CORONA_ sub-study. Data from the third time point (hereafter referred to as T2) were collected between October 2020 and February 2021 as part of the second survey of the DREAM_CORONA_ sub-study. However, we excluded participants who completed the DREAM_CORONA_ questionnaires after a specified time frame when the restrictions were notably adapted (i.e., after June 5, 2020, for T1 and after December 12, 2020, for T2, respectively). While changes in relationship satisfaction were assessed at all three measurement points, predictors and covariates were measured at T0 and T1.

### Sample

**Fig 2:**
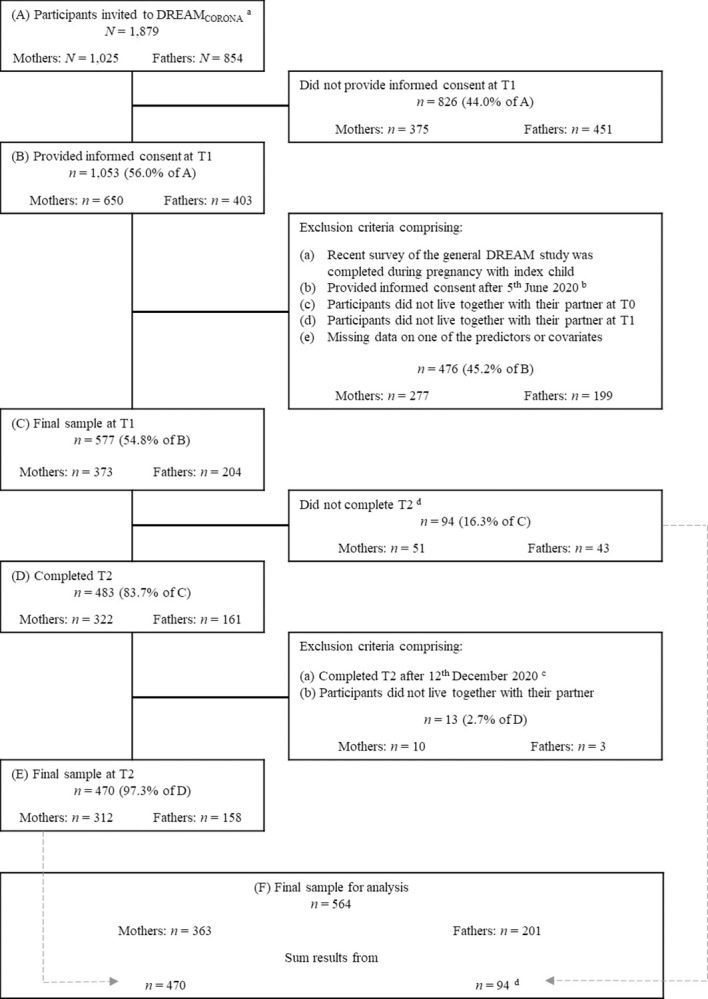
Flowchart of retention rate and exclusion criteria resulting in the final sample. ^a^Online participants of the general DREAM study as of April 2020 (twin and multiple pregnancies excluded). ^b^On June 6, 2020, new regulations came into effect. ^c^After December 12, 2020, a new lockdown started. ^d^Compared to the preregistered analysis plan, it was possible to add another 94 individuals with missing data at T2 to the final sample.

Of the 1,879 participants who were invited to the DREAM_CORONA_ sub-study, 1,053 gave written informed consent to participate. As previously mentioned, we set exclusion criteria regarding the time frame of completing T1 and T2 when the restrictions notably changed. Further exclusion criteria were pregnancy with the index child at T1 of the DREAM_CORONA_ study, being separated from the partner at any measurement point, and missing data on any of the predictors. For the current analyses, the final sample consisted of 564 participants of whom 363 (64.4%) were mothers and 201 (35.6%) were fathers (Fig 2). Of the final sample, 470 participants (83.3%) had completed all measurement points. In the preregistered analysis plan, 94 participants with missing T2 data were incorrectly excluded due to an error in the syntax that was noticed only after the protocol was published. Therefore, the sample size (*N* = 564) includes these 94 more participants than originally planned (*N* = 470). However, to account for possible differences between these two groups of completers and non-completers, we conducted attrition analyses.

### Measures

#### Relationship satisfaction

The German version of the Short Form of the Partnership Questionnaire (PFB-K) was used for parents’ self-reports of relationship satisfaction at all three measurement points. The PFB-K is based on the Partnerschaftsfragebogen (PFB) [80]. It consists of three subscales (conflict behavior, tenderness, communication) with three items each and one item assessing global happiness. The items of the subscales are answered on a scale ranging from 0 (*never/very rarely)* to 3 (*very often)* and the happiness item is rated on a scale from 0 (*very unhappy)* to 5 (*very happy)*. The total score is computed by summing up all items [81]. For this study, only the sum score (range: 0–27) of the nine subscale items of the PFB-K was analyzed, as it has been shown that it correlates very highly with the total score of the PFB [81]. In our sample, the PFB-K showed good reliability (T0: α = .82; T1: α = .82; T2: α = .84).

#### Predictors

*Changes in the division of housework and childcare*. Mothers and fathers were asked to rate the division of housework and childcare from 0 (*I do everything)* to 10 (*my partner does everything)* on two separate items at T0 and T1. The items were taken from the Norwegian Akershus Birth Cohort Study. For the analyses, the responses from T0 and T1 were at first categorized as 1 (*I do more*, comprising values from 0 to 3), 0 (*equal*, comprising values from 4 to 6), and -1 (*my partner does more*, comprising values from 7 to 10), respectively. In a second step, the difference between the T0 and T1 scores was then computed to operationalize the changes in the division of housework and childcare at the beginning of the pandemic. Negative values (-1, -2) represented the person doing more than before the pandemic, values of zero represented no change in the division, and positive values (1, 2) represented the person’s partner doing more than before the COVID-19 pandemic.

To simplify the interpretation and so as not to overload the model, these variables comprising the changes in the division of housework and childcare were categorized again as 1 (*I do more than before the pandemic*, comprising values of -1 and -2), 2 (*no change in the division of housework/childcare*, comprising values of 0), and 3 (*my partner does more than before the pandemic*, comprising values of 1 and 2). For instance, the shift from “my partner does more” at T0 to “equal” at T1 fell in the same category as the shift from “equal” at T0 to “I do more” at T1, i.e., “I do more than before the pandemic”.

*External childcare situation*. Mothers and fathers answered the question “Does your child currently attend a childcare facility (e.g., nursery, kindergarten, or daycare/childminder)?” at T1 with three possible answers: 1 (*yes)*, 2 (*no*, *but before the COVID-19 pandemic)*, and 3 (*no)*. The question was developed based on a similar question from the questionnaire for the Socio-Economic Panel (SOEP) Innovation Sample [82].

*Perceived pandemic-related stress*. Perceived pandemic-related stress was measured at T1 with the question “In general, how burdened do you feel by the current situation regarding the novel coronavirus or COVID-19 and the associated impact (e.g., on work, leisure, childcare, or financial situation)?” on a scale from 1 *(not burdened at all)* to 11 *(very heavily burdened)*.

#### Covariates

*Education*. Education was assessed using a question from the German National Cohort [83] as part of the main DREAM study questionnaire that mothers and fathers completed during pregnancy. The answers were categorized as 0 *(≤ 10 years of schooling)* and 1 (*> 10 years)*.

*Number of children per household*. The question “How many children live in your household?” was asked at T1. For the analyses, it was operationalized as a dichotomous variable with 0 (*one child)* and 1 (*more than one child)*.

*Age of the index child*. The age of the index child in months was assessed at T1.

### Statistical analyses

**Fig 3:**
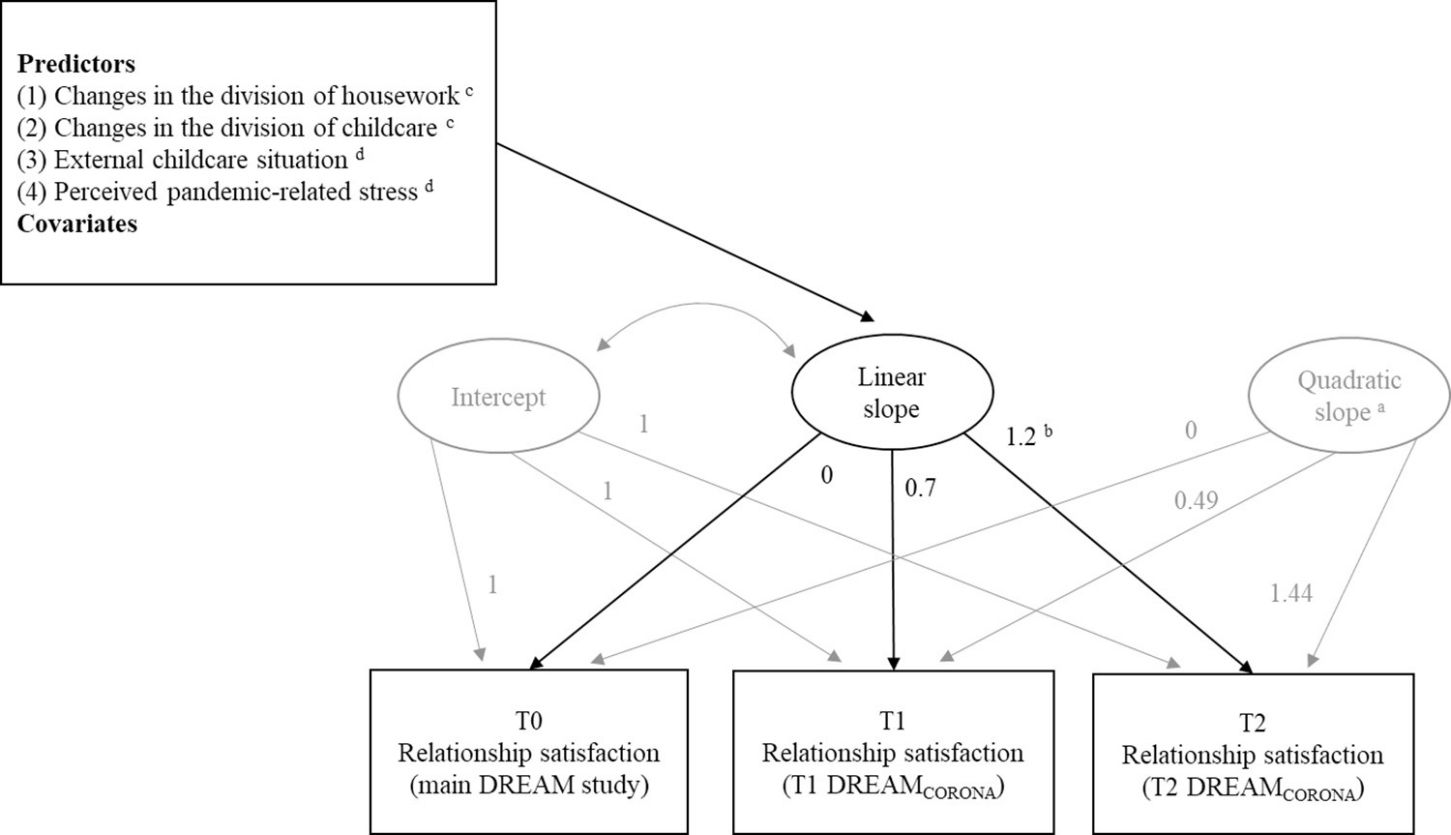
Path diagram for latent variable growth curve model of relationship satisfaction over time. Rectangles represent observed variables. Ellipses represent latent growth factors describing the outcome growth trajectory, defined by time scores. Arrows between the repeatedly measured outcome and the latent growth factors, as well as between the latent growth factors and the predictors and covariates represent linear regression relationships. ^a^The variance of the quadratic slope was constrained to zero. ^b^In the preregistered analysis plan, this fixed slope loading was slightly higher (i.e., 1.3) because it was determined based on a smaller sample size (as 94 individuals with missing data at T2 could be additionally included compared to the preregistered analysis plan). ^c^Change in the division between T0 and T1. ^d^Assessed at T1.

Latent Growth Curve Modelling (LGCM) was used to examine changes in parental relationship satisfaction over time as well as the role of changes in family routines and perceived pandemic-related stress for these changes. In LGCMs, a repeatedly measured outcome variable (relationship satisfaction) is regressed on so-called latent growth factors (intercept, linear slope, quadratic slope, etc.) that represent the trajectory over time (Fig 3). The intervals between measurements are captured by fixed factor loadings. Predictors and covariates are incorporated into LGCMs by regressing the latent growth factors onto these variables. In our case, the growth factors were predicted by changes in the division of housework and childcare, external childcare situation, and perceived pandemic-related stress. We chose LGCM because it offers several advantages [84] over other analytical strategies such as predicting a difference score between two points in time. With LGCMs, it is possible to analyze complex longitudinal data and non-linear changes over time. Developmental trajectories at the group level can be investigated, while individual differences in the trajectories are captured by the variance of the latent growth factors. What is more, missing data are handled by a full-information maximum likelihood (FIML) estimator [85] that uses all available data under a missing at random assumption. Particularly advantageous for our research questions was that LGCMs allow to analyze multiple predictors and covariates of the change of relationship satisfaction over three measurement points. An overview of the planned model can be found in Fig 3.

The analyses were carried out in IBM SPSS Statistics (Version 27.0) and Mplus [Version 7.31; 86]. The data were checked for multicollinearity and outliers. Relationship satisfaction scores that deviated more than three standard deviations from the mean were considered missing. Accordingly, single values were removed for six participants. One person was completely excluded due to highly differing scores at all three measurement points. As planned in our preregistered analysis plan, we conducted all analyses once without and once with outliers. All reported analyses, except for the multicollinearity test, are based on the sample without outliers. The results of the full sample, including outliers and deviating single values, are presented in S1 File. Time intervals between the measurement points showed a great variation (range T0–T1: 2 to 21 months, range T1–T2: 4 to 6 months). Therefore, we calculated every model with fixed slope loadings at average values and with individual time scores and chose the better-fitting model. The fixed slope loadings were determined as follows: 0, 0.7, and 1.2 (unit: 0.1 = 1 month). Model fit was assessed using the Akaike information criterion (AIC) and Bayesian information criterion (BIC). These information criteria balance fit and parsimony of latent models, whereas lower values indicate superior model fit. The following additional fit indices were used for model fit estimation of the models calculated with fixed slope loadings: chi-square (χ2), the root mean square error of approximation (RMSEA), the standardized root-mean-square residual (SRMR), the comparative fit index (CFI), and the Tucker-Lewis index (TLI). Model fit was estimated in detail using the following cut-off values by Hu and Bentler [87]: χ2/df < 3.00, RMSEA ≤ .06, SRMR ≤ .08, CFI ≥ .95, TLI ≥ .95. Comparisons between models with fixed slope loadings and those with individual time scores had to be based on AIC and BIC values only, because these are the only fit indices available for growth models with individual time scores.

To analyze the changes in parental relationship satisfaction, the model was first run without any predictors or covariates for the total sample. The variance of the quadratic growth factor was fixed at zero to ensure model identification. To examine possible differences between mothers and fathers, the model was calculated again as a multi-group model. To test the significance of the differences between the model parameter results for mothers and fathers, a model with equal parameters for both mothers and fathers was compared to the model without parameter restrictions. For that purpose, the Chi-square difference test was used. Next, models were calculated in which the latent growth factors were regressed on the predictors and covariates once for the whole sample and once as a multi-group model for mothers and fathers. The predictors changes in the division of housework and childcare and external childcare situation as well as the covariates education and number of children per household were dummy-coded. Please note that in the preregistered analysis plan, the predictors changes in the division of childcare and housework were meant to be included as ordinally scaled variables rather than as dummy-coded difference scores. This adjustment was made to simplify interpretation.

Maximum likelihood estimation with robust standard errors was used. Prospective associations are given as standardized regression coefficients with 95% confidence intervals. We used the standard *p* < .05 criterion to determine whether the results were statistically significant.

### Attrition analyses

We conducted attrition analyses (tables available on request) comparing participants who completed all three measurement points (*n* = 469; completers) to participants who completed only T0 and T1 (*n* = 94; non-completers) for the following variables: relationship satisfaction at T0 and T1, predictors (changes in division of housework and childcare from T0 to T1, external childcare situation, and perceived pandemic-related stress), covariates (education, number of children per household, and age of index child), and further sociodemographic factors (age and employment). There were no significant differences between completers and non-completers regarding the mentioned variables except for age and division of childcare. More specifically, for both mothers (32.0 vs. 33.4 years; *U* = 6281.00, *z* = -2.24, *p* = .025) and fathers (33.7 vs. 35.2 years; *U* = 2663.00, *z* = -2.18, *p* = .030) the completer group was younger than the non-completer group. Mothers (but not fathers) who completed T2 showed a different pattern in the division of childcare at T1 (*U* = 6709.50, *z* = -2.02, *p* = .043). They were more likely to report no change (completers: 62.1% vs. non-completers: 52.9%) or that their partner did more childcare than before the pandemic (18.6% vs. 13.7%). In contrast, more non-completers (33.3%) than completers (19.3%) reported doing more childcare than before the pandemic. Thus, our attrition analyses suggest selective participation over time which makes the assumption that data are missing at random (MAR) rather than missing completely at random (MCAR) reasonable. This assumption is also supported by Little’s Missing Completely at Random Test [88], *Χ^2^* (12) = 23.22, *p* = .026. It has been shown that the FIML estimator used for the LGCMs in the present study can adequately handle data missing at random [89].

### Ethical statement

This study involving human participants was reviewed and approved by the Ethics Committee of the Technische Universität Dresden (No: EK 278062015). In addition, we have received feedback and approval from the data protection officers of both our faculty and the state of Saxony, and we maintain regular contact with the faculty’s data protection officer on data protection issues. All participants provided written informed consent to participate in this study. They were assured of the pseudonymization of their data as well as the possibility to terminate the study at any time without disadvantage. Additional information regarding the ethical, cultural, and scientific considerations specific to inclusivity in global research is included in the S3 File.

## Results

### Descriptive statistics

Descriptive statistics are presented in Tables 1 and 2. The sample consisted mainly of highly educated parents, with 85.1% having more than 10 years of school education (see Table 1). The average age of the mothers was 32.2 years (*SD* = 3.8) and of the fathers 34.0 years (*SD* = 5.0). Most of them (76.2%) had only one child, with the number of children ranging from one to three. Most of the fathers were employed at T1 (89.1%), whereas more than half of the mothers (55.2%) were not. Among those who were not currently employed, the reasons were maternity leave or pregnancy-related employment ban for 91.0% of the mothers and paternity leave for about half of the fathers (45.5%). Of the total sample, 18.8% of the participants reported that their children were not in external childcare due to the COVID-19 pandemic. Both before and at the beginning of the pandemic, more than half of the total sample reported sharing housework (T0: 63.2%; T1: 63.6%) and childcare (T0: 52.8%; T1: 49.2%) equally (see Table 2). Regarding the changes in role division, 21.0% of the mothers and 13.4% of the fathers reported doing more housework, and 21.3% of the mothers and 28.4% of the fathers reported doing more childcare than before the pandemic.

**Table 1:** Descriptive statistics.

	Total sample	Mothers	Fathers
	*n* = 563	*n* = 362	*n* = 201
**Age (in years)**^a^, *M (SD)*	32.8 (4.4)	32.2 (3.8)	34.0 (5.0)
**Employment**^a^^,^^b^, *n* (%)			
Yes	341 (60.6%)	162 (44.8%)	179 (89.1%)
No	222 (39.4%)	200 (55.2%)	22 (10.9%)
**Current paternity/maternity leave**^c^ **among those currently not employed (*n* = 222)** ^a^, *n* (%)			
Yes	192 (86.5%)	182 (91.0%)	10 (45.5%)
No	30 (13.5%)	18 (9.0%)	12 (54.5%)
**Education**^d^, *n* (%)			
≤ 10 years	84 (14.9%)	49 (13.5%)	35 (17.4%)
> 10 years	479 (85.1%)	313 (86.5%)	166 (82.6%)
**Number of children per household**^a^, *n* (%)			
One child	429 (76.2%)	271 (74.9%)	158 (78.6%)
More than one child	134 (23.8%)	91 (25.1%)	43 (21.4%)
**Age of the index child** (in months)^a^, min-max	3–34	3–34	3–32
**External childcare situation**^a^, *n* (%)			
Yes	180 (32.0%)	109 (30.1%)	71 (35.2%)
No, but before the COVID-19 pandemic	106 (18.8%)	74 (20.4%)	32 (15.9%)
No	277 (49.2%)	179 (49.4%)	98 (48.8%)
**Perceived pandemic-related stress**^a^, *M (SD)*	5.60 (2.66)	5.82 (2.65)	5.14 (2.63)
**Relationship satisfaction**^e^, *M (SD)*			
T0	19.48 (4.35)	19.62 (4.46)	19.21 (4.13)
T1	18.95 (4.42)	19.01 (4.50)	18.85 (4.28)
T2	19.02 (4.65)	18.97 (4.80)	19.11 (4.36)

*n* might vary due to missing data. Data were collected at T0 (pre-pandemic, i.e., August 2018–March 2020), T1 (May–June 2020), and T2 (October–December 2020).

^a^Assessed at T1.

^b^Employment comprises: working full-time or part-time, irregular or marginal employment, being in an apprenticeship, or doing a voluntary service.

^c^For mothers comprising: maternity leave or pregnancy-related employment ban.

^d^Assessed at the first survey of the main DREAM study.

^e^Sum score of the Short Form of the Partnership Questionnaire (range: 0 to 27).

**Table 2:** Descriptive statistics for division of housework and childcare before and at the early stage of the COVID-19 pandemic.

	Total sample		Mothers		Fathers	
	*n* = 563		*n* = 362		*n* = 201	
	T0	T1	T0	T1	T0	T1
**Division of housework**, *n* (%)						
I do more	130 (23.1%)	139 (24.7%)	103 (28.4%)	121 (33.4%)	27 (13.4%)	18 (9.0%)
Equal	356 (63.2%)	358 (63.6%)	225 (62.2%)	222 (61.3%)	131 (65.2%)	136 (67.6%)
My partner does more	77 (13.7%)	66 (11.7%)	34 (9.4%)	19 (5.3%)	43 (21.4%)	47 (23.4%)
**Division of childcare**, *n* (%)						
I do more	152 (27.0%)	179 (31.8%)	147 (40.6%)	163 (45.0%)	5 (2.5%)	16 (8.0%)
Equal	297 (52.8%)	277 (49.2%)	204 (56.4%)	180 (49.7%)	93 (46.3%)	97 (48.2%)
My partner does more	114 (20.2%)	107 (19.0%)	11 (3.0%)	19 (5.3%)	103 (51.2%)	88 (43.8%)
	**From T0 to T1**		**From T0 to T1**		**From T0 to T1**	
**Changes in the division of housework**^a^, *n* (%)						
I do more than before the pandemic	103 (18.3%)		76 (21.0%)		27 (13.4%)	
No change	373 (66.3%)		238 (65.7%)		135 (67.2%)	
My partner does more than before the pandemic	87 (15.5%)		48 (13.3%)		39 (19.4%)	
**Changes in the division of childcare**^a^, *n* (%)						
I do more than before the pandemic	134 (23.8%)		77 (21.3%)		57 (28.4%)	
No change	332 (59.0%)		220 (60.8%)		112 (55.7%)	
My partner does more than before the pandemic	97 (17.2%)		65 (18.0%)		32 (15.9%)	

*n* might vary due to missing data. Data were collected at T0 (pre-pandemic, i.e., August 2018–March 2020) and T1 (May–June 2020).

^a^Change in the division between T0 and T1. These variables were used in the main analyses.

### Multicollinearity

For the total sample, there were six statistically significant correlations between the predictors and covariates (see Table 3). The multicollinearity analysis revealed high correlations between the age of the index child and the external childcare situation. Therefore, the age of the index child was omitted from the following analyses.

**Table 3:** Correlations between predictors and covariates for the total sample.

Predictors/covariates	1^a^	2^a^	3^a^	4	5	6	7
1. Changes in the division of housework^a^^,^^b^	–						
2. Changes in the division of childcare^a^^,^^b^	.18**	–					
3. External childcare situation^a^^,^^c^	.03	.01	–				
4. Perceived pandemic-related stress^c^	-.06	.01	-.21**	–			
5. Education^d^	.00	.03	.05	.01	–		
6. Number of children per household^c^	.01	.04	-.09*	.20**	.01	–	
7. Age of the index child^c^	-.03	-.00	-.55**	.27**	.03	.17**	–

Pearson correlation coefficients are reported. Data were collected at T0 (pre-pandemic, i.e., August 2018–March 2020), T1 (May–June 2020), and T2 (October–December 2020).

* *p* < .05

** *p* < .01

^a^Kendall’s τ correlation coefficient.

^b^Change in the division between T0 and T1.

^c^Assessed at T1.

^d^Assessed at the first survey of the main DREAM study.

### Changes in relationship satisfaction during the COVID-19 pandemic

To examine the changes in the relationship satisfaction for the total sample, the LGCM was calculated using individual time scores (Model 1) because this resulted in a better model fit than using the average slope loadings (see Table 4). Model results showed a small, albeit non-significant decline in relationship satisfaction, as indicated by a negative linear slope of -.54 and a quadratic slope close to zero (Table 5). Sensitivity analyses with outliers included yielded similar findings (see S1 File).

**Table 4:** Comparison of model fit.

	LGCM with average slope loadings		LGCM with individual time scores	
	AIC	BIC^a^	AIC	BIC^a^
**Unconditional models**				
Model 1: Total sample	8290.59	8301.01	8288.53	8298.96
Model 2: Multi-group model	8296.25	8317.11	8296.41	8317.27
Model 3: Multi-group model with zero variance for the quadratic growth factor	8297.91	8314.13	8294.06	8310.29
**Models with predictors and covariates**				
Model 4: Total sample	8296.61	8316.31	8293.56	8313.26
Model 5: Multi-group model	8306.18	8345.58	8323.29	8362.69

^a^Sample-size adjusted BIC (n* = (*n* + 2) / 24).

**Table 5:** LGCM results.

		Linear change in relationship satisfaction			Quadratic change in relationship satisfaction		
		Mean^a^	95% CI	*p*	Mean	95% CI	*p*
**Unconditional models**							
Model 1: Total sample		-.54	[-1.28, 0.21]	.156	.03	[-0.53, 0.59]	.921
Model 3	Mothers	-.51	[-1.46, 0.45]	.296	-.11	[-0.83, 0.61]	.770
	Fathers	-.65	[-1.74, 0.45]	.247	.32	[-0.45, 1.10]	.413
**Models with predictors and covariates**							
Model 4: Total sample		-.19	[-1.36, 0.98]	.749	.04	[-0.51, 0.60]	.888
Model 5	Mothers	-.23	[-1.82, 1.38]	.783	.67	[-0.14, 1.48]	.106
	Fathers	-.97	[-2.97, 1.02]	.339	.70	[-0.48, 1.88]	.242

^a^For the LGCMs with predictors, the intercept of the linear slope is given instead of the mean.

### Differences between mothers and fathers

For the multi-group model, the model with average slope loadings reached a better model fit at first (Model 2). However, the model could not be estimated properly because the latent variable covariance matrix was not positive definite in the male group, due to a negative variance of the linear slope. Solving this issue by fixing the linear slope variance at zero did result in a better model fit for the model using individual time scores (Model 3). Therefore, for the multi-group model without predictors, we decided to use the LGCM model with individual time scores, as there were no restrictions on the slope variance necessary (see Table 4). The multi-group analysis showed no significant change in the relationship satisfaction over time for neither mothers nor fathers (see Model 3 results in Table 5). There were no significant differences between the multi-group model with slopes constrained to be equal for both groups and the one with no parameter constraints (*χ2* (1, 563) = 0.04; *p* = .838) indicating that mothers and fathers did not differ in their changes in relationship satisfaction. Sensitivity analyses showed that when outliers were included, there was also no significant change in the relationship satisfaction over time for neither mothers nor fathers (see S1 File (1)).

### Predictors of the changes in relationship satisfaction during the COVID-19 pandemic

For the total sample and when the predictors were included (Model 4), the model with individual time scores provided a better model fit than the model with average slope loadings (see Table 4). Compared to no change in the division of housework during the pandemic, doing more housework showed a negative association with the changes in relationship satisfaction (*b* = -.63; CI = [-1.27, -0.00]; *p* = .051; see Table 6), although only marginally significant. In addition, although the p-value was exactly .05 due to rounding, we found that feeling burdened by the COVID-19 pandemic (i.e., predictor perceived pandemic-related stress) appeared to be negatively associated with the trajectory of relationship satisfaction over time (*b* = -.09; CI = [-0.19, -0.00]; *p* = .050; see Table 6).

**Table 6:** Prospective associations between predictors and linear change in relationship satisfaction while adjusting for covariates.

	Linear change in relationship satisfactionb										
Predictors	Model 4: Total sample			Model 5: Mothers				Model 5: Fathers			
	*b*	95% CI	*p*	*b*	ß^a^	95% CI	*p*	*b*	ß^a^	95% CI	*p*
**Changes in the division of housework**^c^ (Ref.: No change)											
I do more than before the pandemic	-.63	[-1.27, -0.00]	.051	**-1.06**	**-.67**	**[-1.22, -0.13]**	**.016**	.48	.51	[-0.64, 1.66]	.388
My partner does more than before the pandemic	-.00	[-0.61, 0.61]	.997	-.57	-.36	[-0.92, 0.20]	.204	.50	.53	[-0.65, 1.70]	.378
**Changes in the division of childcare**^c^ (Ref.: No change)											
I do more than before the pandemic	.18	[-0.41, 0.77]	.546	-.37	-.23	[-0.77, 0.30]	.389	.55	.58	[-0.51, 1.67]	.301
My partner does more than before the pandemic	.23	[-0.40, 0.85]	.477	**.86**	**.54**	**[0.05, 1.04]**	**.032**	-.38	-.40	[-1.71, 0.91]	.552
**External childcare situation**^d^ (Ref.: No)											
Yes	.30	[-0.21, 0.81]	.252	-.10	-.07	[-0.53, 0.40]	.783	.54	.56	[-0.56, 1.69]	.328
No, but before the COVID-19 pandemic	.44	[-0.24, 1.12]	.202	-.05	-.03	[-0.57, 0.51]	.916	.99	1.04	[-0.71, 2.80]	.245
**Perceived pandemic-related stress** ^d^	**-.09**	**[-0.19, -0.00]**	**.050**	-.11	-.18	[-0.39, 0.03]	.100	-.08	-.21	[-0.83, 0.42]	.515
**Education**^e^ (Ref.: ≤ 10 years)											
> 10 years	-.10	[-0.77, 0.60]	.781	-.40	-.25	[-0.86, 0.36]	.417	-.28	-.29	[-1.41, 0.82]	.606
**Number of children per household**^d^ (Ref.: One child)											
More than one child	.39	[-0.12, 0.90]	.131	.40	.25	[-0.19, 0.69]	.264	-.01	-.01	[-0.80, 0.78]	.974

*b* = unstandardized coefficients, ß = standardized coefficients. Data were collected at T0 (pre-pandemic, i.e., August 2018–March 2020), T1 (May–June 2020), and T2 (October–December 2020).

^a^Standardization was different for binary (*b*/*SD* (slope)) and continuous variables (b*SD (predictor)/*SD* (slope)).

^b^Across T0, T1, and T2.

^c^Change in the division between T0 and T1.

^d^Assessed at T1.

^e^Assessed at the first survey of the main DREAM study

Sensitivity analyses with outliers included (see S1 File) showed that perceived pandemic-related stress was no longer a significant predictor for changes in relationship satisfaction for the total sample (*b* = -.07; CI = [-.017, 0.02]; *p* = .145; see S1 File (2)). Still, the negative association of doing more housework with changes in relationship satisfaction was also present when outliers were included.

#### Differences between mothers and fathers

For the multi-group model with the predictors and covariates included (Model 5), using the average slope loadings resulted in a better model fit (see Table 4). The model reached a good model fit (χ2/df = 2.05, RMSEA = 0.06, SRMR = .05, CFI = .95, TLI = .92). For fathers, there were no statistically significant associations between the predictors and their relationship satisfaction over time. If mothers did more housework than before the pandemic, this had a high negative association with their relationship satisfaction over time (*ß* = -.67; CI = [-1.22, -0.13]; *p* = .016; see Table 6). Additionally, reporting that their partner did more childcare than before the pandemic was associated with improved relationship satisfaction of mothers (*ß* = .54; CI = [0.05, 1.04]; *p* = .032; see Table 6). The multi-group model with equal slopes and regression coefficients for mothers and fathers and the one with freely estimated values were not significantly different from each other (*χ2* (12, 563) = 19.96; *p* = .068). All results were similar within sensitivity analyses regarding outliers (see S1 File).

## Discussion

### Summary of findings

This study, based on a preregistered analysis plan, examined the changes in relationship satisfaction among young parents in Germany from pre-pandemic times to the second wave in December 2020, as well as the role of changes in family routines (i.e., division of housework and childcare and the availability of external childcare facilities) and perceived pandemic-related stress during the early stages of the pandemic, while adjusting for education and number of children per household. Relationship satisfaction remained stable over time for the total sample as well as when examining mothers and fathers separately. Changes in the division of housework and childcare, and perceived pandemic-related stress showed meaningful associations with the changes in relationship satisfaction over time.

### Changes in relationship satisfaction during the COVID-19 pandemic

Contrary to our hypothesis, relationship satisfaction remained stable over time, suggesting that there was no general change in relationship satisfaction among young parents from pre-pandemic times up to the first year of the COVID-19 pandemic. This finding is in line with results from the beginning of the pandemic [12, 13], but also contrasts with other literature that found a decline in relationship quality over the course of the COVID-19 pandemic [9, 16, 17]. However, the studies that found a decline in relationship quality [9, 16, 17] used retrospective questions to examine the relationship quality before the pandemic or asked directly about pandemic-related changes. These methods create a more suggestive framework compared to longitudinal and prospective study designs and could have led to stronger associations. In addition to methodological reasons, the discrepant findings may also arise from different populations. For instance, a study that also used longitudinal data from a German population reported a decline in relationship satisfaction [15]. However, it should be noted that this study was conducted on a population of employed individuals, not all of whom were parents. Conversely, our study focused solely on parents of young children (mostly first-time parents), of whom only 60.6% were employed at the time of the study. In correspondence to our findings, a longitudinal study, which included a distribution of employed and currently non-working participants (63.0% vs. 37.0%) comparable to ours, also found no changes in relationship satisfaction [12]. However, half of the non-working fathers and most non-working mothers in our sample fell into the category of those currently on paternity/maternity leave or pregnancy-related employment ban. Thus, while many of our participants are presumed to be employed or even dual-earner parents, the study’s timing–during the transition to parenthood, when most German mothers interrupt their employment for one to even two years–limits the possibility of focusing on work factors. Still, work-family conflict seemed to have increased due to the COVID-19 pandemic, particularly for employed parents [37], which in turn was found to be negatively associated with relationship quality [90]. Therefore, it would be interesting to further examine the role of employment situation and work-family conflict, especially in conjunction with the division of housework and childcare [91–93], in future research on the relationship satisfaction of working parents.

Contrary to our assumptions, no differences were found between mothers and fathers in the trajectory of their relationship satisfaction. Several studies support this finding [12, 20, 94], but there is also evidence from a Belgian study comprising both parents and childless couples that women reported lower relationship quality than men during the lockdown in April 2020 [30]. Differences in stress experience during the pandemic might have contributed to differences in relationship satisfaction between women and men, as external stressors play a large role for relationship satisfaction [1, 10, 14]. While recent evidence suggests that women may have been more emotionally affected by the pandemic [31, 32], in our sample, women and men scored quite similarly in terms of perceived pandemic-related stress, which may explain the lack of sex differences in relationship satisfaction.

### Predictors of changes in relationship satisfaction of young parents during the COVID-19 pandemic

The prospective associations between changes in family routines (i.e., changes in the division of housework and division of childcare and the external childcare situation), and perceived pandemic-related stress during the early stage of the pandemic, and changes in relationship satisfaction up to the second wave of the COVID-19 pandemic were examined in an exploratory manner. Although our study results suggest that, fortunately, the COVID-19 pandemic was not generally related to changes in young parents’ relationship satisfaction, we did find some notable associations with pandemic-related predictors.

First, perceived pandemic-related stress was negatively associated with parental relationship satisfaction over time, albeit this finding was based on a *p*-value of exactly .05 and could not be replicated in our sensitivity analyses including outliers. However, this finding is consistent with former research showing that stress increased, especially for parents [69], while relationship well-being deteriorated [1, 10, 37]. Therefore, our results highlight the importance of further research into parents’ stress experience during the pandemic and its potentially harmful consequences.

Moreover, there were some meaningful results regarding the changes in the division of housework and childcare from pre-pandemic times to the early stages of the pandemic, specifically for mothers. Reporting doing more housework than before the pandemic appeared to be related to a decline in relationship satisfaction among mothers. This finding is consistent with the results of a study showing that a greater share of housework increases relationship problems and decreases relationship satisfaction for women [64]. Even though this was evident for our whole sample, it did not apply to fathers in the multi-group model, which is in line with previous studies [62, 65]. Additionally, the results of the aforementioned study [64] showed that the association may be related to being the primary caregiver. Fathers who were not employed and took over most of the housework also showed a decrease in relationship satisfaction when they had to take on even more due to the COVID-19 pandemic. Although we did not assess it directly, with 89.1% of the fathers and only 46.1% of the mothers being employed in our sample, we assume that mothers were the primary caregivers more often, which may be an explanation for the sex differences in the association between housework and relationship satisfaction.

For the division of childcare, our results suggest that reporting that their partner was doing more childcare than before the pandemic was beneficial for mothers’ relationship satisfaction. This finding is in line with pre-pandemic studies suggesting that fathers’ involvement in childcare strengthens couple relationship satisfaction [67, 68]. The fact that this did not apply for fathers in the current study is again consistent with findings that the association between relationship satisfaction and childcare may only be relevant for the primary caregiver [64].

Based on the assumption that, overall, women still do most of the housework and childcare [58, 95], our results may suggest that a more equal division would be beneficial for parents. This appears particularly crucial during early parenthood because an unequal division of housework is exacerbated during the transition to parenthood [49–52], which our study population of mainly first-time parents recently experienced. The benefit is suggested by the positive association found between the maternal ratings of their partners doing more childcare than before the pandemic and maternal relationship satisfaction, as well as the negative association found between maternal ratings of they themselves doing even more housework than before the pandemic and their relationship satisfaction. Interestingly, although it was not one of our key variables, the covariate number of children per household showed no meaningful association with relationship satisfaction. Given that more children imply more time for childcare and housework and presumably less time for relationships, we found this surprising and it also contradicts pre-pandemic [77] and COVID-19-related findings [96], which indicate a negative association between the number of children and relationship satisfaction. The absence of an effect in our study may be attributed to the diverging variability of the number of children between the studies. That is, in the latter study, parents had a minimum of two and a maximum of five children, whereas in our sample, most parents had only one child (76.2%) with a maximum number of three children [96].

Regarding the link between the change in external childcare and relationship satisfaction, we did not find any meaningful results in our sample. Thus, parents whose children were cared for externally before the pandemic and who now had to care for them at home did not differ in their relationship satisfaction from parents whose external childcare situation did not change due to the pandemic. It should be noted that the group of parents who reported that their children did not attend external childcare because of the COVID-19 pandemic was rather moderate (18.8%) and it was not possible to ascertain whether this decision was voluntary or not. In the future, it would be valuable to investigate the association between changes in the external childcare situation and parental relationship satisfaction in dual-earner couples after parental leave has run out, to whom our results are not directly applicable. The increased family-work conflict experienced by employed parents in particular due to COVID-19 restrictions [37] could potentially play a role in this, given its negative association with relationship quality [90].

### Strengths and limitations

Our study contributes to the growing body of evidence regarding the changes in relationship satisfaction during the COVID-19 pandemic [for an overview see 11] by highlighting the special situation of parents during the pandemic. Our study, based on a preregistered analysis plan, is one of the first to take a closer look at the prospective associations between changes in family routines with the onset of the pandemic and changes in relationship satisfaction [for another example see 64]. A further strength comprises the prospective longitudinal study design extending beyond the first few months of the pandemic, enabling monitoring of changes over a longer period without relying on retrospective questions. Additionally, the differential consideration of mothers and fathers is an advantage of this study and the DREAM study in general. Moreover, a methodological strength of our analyses lies in the use of individual time scores that allow more precise modeling of the growth curve as the individual time intervals of each participant are observed.

Our study revealed some important results about changes in young parents’ relationship satisfaction during the first year of the COVID-19 pandemic. Nevertheless, the following limitations should be noted. The generalizability of our results is limited by the predominantly high educational status and low employment rate among participants, attributable to ongoing parental leave. It should also be noted that our population mainly consists of first-time parents who may have recently experienced a period of change in the division of housework due to the recent transition to parenthood, as we already know from pre-pandemic studies [49–52]. However, to disentangle the pandemic-related changes from the frequently observed, normative course after childbirth, we excluded participants who completed the surveys during the pregnancy with the index child from our final sample. Still, the findings should be applied with caution to less educated samples as well as to parents of older children or who (both) currently work (i.e., especially mothers who either do not take maternity leave at all or who take it for a shorter duration). Additionally, social desirability in the response behavior can always affect the self-report of relationship satisfaction and the division of housework or childcare. Regarding the beneficial effect of mothers reporting that their partners are doing more childcare, it is important to note that mothers who dropped out of the study at T2 showed this pattern of increased paternal involvement less often than the completers. Therefore, the effect could be slightly overestimated. Lastly, we acknowledge that there is considerable variance within the three categories we used to operationalize changes in the division of housework and childcare (“I do more than before the pandemic”, “my partner does more”, and “equal”). For example, among those who reported before the pandemic (at T0) that their partner did more housework, we could not differentiate between those who then shared the housework equally or did more housework themselves at the beginning of the pandemic (T1). This was decided to simplify the interpretation and not overload the model. However, this could be the starting point for future research to shed light on the dynamics in more detail.

### Future research and practical implications

Our findings highlight the importance of exploring the prospective associations of changes in daily family routines and pandemic-related stress on young parents’ relationship satisfaction. Further research on this topic should investigate in more detail which circumstances strengthened or weakened parental relationship satisfaction throughout the COVID-19 pandemic. To ensure greater certainty that the observed associations are specifically related to the COVID-19 pandemic, it is imperative to account for further potential confounding factors and predictors–such as mental health [97], working conditions [37, 91], financial situation [98], among others–by incorporating longitudinal and pre-pandemic data. In addition, it could be interesting to include child-related factors such as parent-child-bonding and child development in the research process. To examine differential effects, parents should be compared to non-parents and women to men. Also, as we were specifically interested in how changes in the parental role division during the early stages of the COVID-19 pandemic were prospectively associated with changes in relationship over time, we only included predictors that preceded in time, i.e., changes from pre-pandemic times to the early stages of the pandemic. Given the rapid changes in government policies during the pandemic, division of childcare and housework work as well as pandemic-related stress may have changed in the further course of the pandemic [e.g., 59, 91]. Further, this study was based on data from the first year of the pandemic. Therefore, it would also be instructive to examine subsequent developments not only during the further course but also after the pandemic was declared to be contained to derive insights for handling future public health crises.

Although further research is warranted, some practical implications can be drawn from our results. First, the healthcare system should provide counseling services for parents, even or particularly in times of social crisis, as pandemic-related stress seems to have negative consequences on parental relationship satisfaction in highly burdened individuals. Based on the assumption that mothers still do more housework and childcare on average [57, 58, 95], our findings suggest that a more equal division of housework and childcare may be beneficial for parental relationship satisfaction [67, 99], especially for mothers, and presumably also for the family climate as a whole. It would therefore be desirable to promote an equal distribution of household responsibilities between parents and to establish correspondingly supportive conditions in the working environment. Since parental relationship satisfaction is associated with parental mental health [72, 74], general life satisfaction [100], and even child development [75], this issue should be of great interest to social and healthcare systems.

## Conclusion

In summary, our research contributes to the growing literature on the changes in relationship satisfaction during the COVID-19 pandemic by focusing on the special situation of parents. While mothers’ and fathers’ relationship satisfaction fortunately did not decrease significantly over the observed period until November 2020, our exploratory analyses revealed intriguing associations between relationship satisfaction and perceived pandemic stress and changes in the division of housework and childcare. Perceived pandemic-related stress seemed to be negatively associated with parental relationship satisfaction. For mothers, doing more housework than before the pandemic appeared to be detrimental to their relationship satisfaction. In addition, reporting that their partner takes on more childcare than before the pandemic seems to be beneficial for mothers’ relationship satisfaction. Considering the exploratory nature of our analyses, these findings should be regarded as a foundation for further promising research endeavors and the formulation of hypotheses.

## Supporting information

S1 File Results with outliers included. (DOCX)

S2 File STROBE statement. (DOCX)

S3 File Inclusivity in global research. (DOCX)
