# Supplementary material for: Relationship satisfaction and family routines of young parents before and during the first year of the COVID-19 pandemic: A latent growth curve analysis
Source: PLoS One. 2024 Feb 16;19(2):e0297740. doi: 10.1371/journal.pone.0297740 (PMC10871525; doi:10.1371/journal.pone.0297740)
Supplement: S1 File — (DOCX) [file pone.0297740.s001.docx]

# S1 File. Results with the outliers included.

**S1 (1) Table. LGCMs results (with outliers included).**

|  | | **Linear change in relationship satisfaction** | | | **Quadratic change in relationship satisfaction** | | |
| --- | --- | --- | --- | --- | --- | --- | --- |
|  | | Mean | 95% CI | *p* | Mean | 95% CI | *p* |
| **Unconditional models** | |  |  |  |  |  |  |
| Model S1: Total sample | | -.56 | [-1.31, 0.19] | .144 | .03 | [-0.52, 0.59] | .914 |
| Model S2 | Mothers | -.55 | [-1.52, 0.42] | .264 | -.08 | [-0.80, 0.63] | .818 |
|  | Fathers | -.61 | [-1.70, 0.48] | .273 | .27 | [-0.51, 1.05] | .492 |
| **Models with predictors and covariates** | |  |  |  |  |  |  |
| Model S3: Total sample | | -.28 | [-1.45, 0.89] | .638 | .03 | [-0.52, 0.58] | .915 |
| Model S4 | Mothers | -.24 | [-1.84, 1.36] | .770 | .60 | [-0.20, 1.40] | .140 |
|  | Fathers | -1.08 | [-3.08, 0.92] | .291 | .67 | [-0.49, 1.83] | .259 |

For all models, the variance of the quadratic slope was fixed at zero. For the models S3 and S4, the intercept of the linear slope is given instead of the mean.

**S1 (2) Table. Prospective associations of the predictors on the linear change in relationship satisfaction (with outliers included) while adjusting for covariates.**

| **Predictors** | **Linear change in relationship satisfaction**^b^ | | | | | | | | | | |
| --- | --- | --- | --- | --- | --- | --- | --- | --- | --- | --- | --- |
|  | **Model S3: Total sample** | | | **Model S4: Mothers** | | | | **Model S4: Fathers** | | | |
|  | *b* | 95% CI | *p* | *b* | ß^a^ | 95% CI | *p* | *b* | ß^a^ | 95% CI | *p* |
| **Changes in division of housework**^c^ (Ref.: No change) |  |  |  |  |  |  |  |  |  |  |  |
| I do more | **-.68** | **[-1.33, -0.03]** | **.039** | **-1.14** | **-.72** | **[-1.29, -0.15]** | **.014** | .54 | .46 | [-0.38, 1.30] | .287 |
| My partner does more | .11 | [-0.51, 0.73] | .731 | -.41 | -.26 | [-0.83, 0.31] | .376 | .55 | .47 | [-0.42, 1.35] | .300 |
| **Changes in division of childcare**^c^ (Ref.: No change) |  |  |  |  |  |  |  |  |  |  |  |
| I do more | .26 | [-0.33, 0.85] | .393 | -.35 | -.22 | [-0.77, 0.33] | .435 | .69 | .58 | [-0.21, 1.37] | .150 |
| My partner does more | .32 | [-0.31, 0.95] | .313 | **.98** | **.62** | **[0.11, 1.13]** | **.017** | -.29 | -.25 | [-1.28, 0.79] | .640 |
| **External childcare situation**^d^  (Ref.: No change) |  |  |  |  |  |  |  |  |  |  |  |
| Yes | .28 | [-0.24, 0.81] | .293 | -.07 | -.04 | [-0.51, 0.42] | .855 | .39 | .33 | [-0.49, 1.15] | .426 |
| No, but before the COVID-19 pandemic | .30 | [-0.43, 1.03] | .416 | -.29 | -.18 | [-0.78, 0.42] | .554 | .94 | .80 | [-0.41, 2.00] | .196 |
| **Perceived pandemic-related stress**^d^ | -.07 | [-0.17, 0.02] | .145 | -.08 | -.13 | [-0.35, 0.08] | .217 | -.06 | -.12 | [-0.56, 0.32] | .587 |
| **Education**^e^ (Ref.: ≤ 10 years) |  |  |  |  |  |  |  |  |  |  |  |
| > 10 years | -.14 | [-0.82, 0.54] | .683 | -.41 | -.26 | [-0.87, 0.35] | .400 | -.30 | -.25 | [-1.13, 0.62] | .571 |
| **Number of children per household**^c^  (Ref.: One child) |  |  |  |  |  |  |  |  |  |  |  |
| More than one child | .32 | [-0.24, 0.88] | .257 | .25 | .16 | [-0.36, 0.67] | .547 | .05 | .04 | [-0.60, 0.68] | .905 |

*b* = unstandardized coefficients, ß = standardized coefficients. Data were collected at T0 (pre-pandemic, i.e., August 2018–March 2020), T1 (May–June 2020), and T2 (October–December 2020).

^a^Standardization was different for binary (*b*/*SD* (slope)) and continuous variables (*b***SD* (predictor)/*SD* (slope)).

^b^Across T0, T1, and T2.
^c^Change in the division between T0 and T1.

^d^Assessed at T1.

^e^Assessed at the first survey of the main DREAM study.
