# Supplementary material for: Relationship satisfaction and family routines of young parents before and during the first year of the COVID-19 pandemic: A latent growth curve analysis
Source: PLoS One. 2024 Feb 16;19(2):e0297740. doi: 10.1371/journal.pone.0297740 (PMC10871525; doi:10.1371/journal.pone.0297740)
Supplement: S2 File — (DOCX) [file pone.0297740.s002.docx]

# S2 File. STROBE Statement.

STROBE Statement—checklist of items that should be included in reports of observational studies

|  | Item No. | Recommendation | Page  No. | Relevant text from manuscript |
| --- | --- | --- | --- | --- |
| **Title and abstract** | 1 | (*a*) Indicate the study’s design with a commonly used term in the title or the abstract | 1  2-3 | Title: Relationship satisfaction and family routines of young parents before and during the first year of the COVID-19 pandemic: A latent growth curve analysis  Abstract |
|  |  | (*b*) Provide in the abstract an informative and balanced summary of what was done and what was found | 2-3 | Abstract |
| Introduction | | | |  |
| Background/rationale | 2 | Explain the scientific background and rationale for the investigation being reported | 3-9 | Introduction |
| Objectives | 3 | State specific objectives, including any prespecified hypotheses | 9 | Introduction (ll. 204-215) |
| Methods | | | |  |
| Study design | 4 | Present key elements of study design early in the paper | 7-8  11 | Methods – Study design (ll. 239-250)  Fig 1. Used surveys of the main DREAM study (T0) and the DREAM_CORONA_ sub-study (T1, T2) |
| Setting | 5 | Describe the setting, locations, and relevant dates, including periods of recruitment, exposure, follow-up, and data collection | 9-10 | Methods – Study design |
| Participants | 6 | (*a*) *Cohort study*—Give the eligibility criteria, and the sources and methods of selection of participants. Describe methods of follow-up  *Case-control study*—Give the eligibility criteria, and the sources and methods of case ascertainment and control selection. Give the rationale for the choice of cases and controls  *Cross-sectional study*—Give the eligibility criteria, and the sources and methods of selection of participants | 9-10  11  12 | Methods – Study design  Methods – Sample  Fig 2. Flowchart of retention rate and exclusion criteria resulting in the final sample |
|  |  | (*b*) *Cohort study*—For matched studies, give matching criteria and number of exposed and unexposed  *Case-control study*—For matched studies, give matching criteria and the number of controls per case |  |  |
| Variables | 7 | Clearly define all outcomes, exposures, predictors, potential confounders, and effect modifiers. Give diagnostic criteria, if applicable | 12-14 | Methods – Measures |
| Data sources/ measurement | 8* | For each variable of interest, give sources of data and details of methods of assessment (measurement). Describe comparability of assessment methods if there is more than one group | 12-14 | Methods – Measures |
| Bias | 9 | Describe any efforts to address potential sources of bias | 17  33-34 | Methods – Attrition analyses  Discussion (ll. 693-715) |
| Study size | 10 | Explain how the study size was arrived at | 11-12 | Methods – Sample, including Fig 2. Flowchart of retention rate and exclusion criteria resulting in final sample |
| Quantitative variables | 11 | Explain how quantitative variables were handled in the analyses. If applicable, describe which groupings were chosen and why | 12-14  14-17 | Methods – Measures  Methods – Statistical analyses |
| Statistical methods | 12 | (*a*) Describe all statistical methods, including those used to control for confounding | 14-17 | Methods – Statistical analyses |
|  |  | (*b*) Describe any methods used to examine subgroups and interactions | 14-17 | Methods – Statistical analyses |
|  |  | (*c*) Explain how missing data were addressed | 14 | Methods – Statistical analyses (ll. 344-346) |
|  |  | (*d*) *Cohort study*—If applicable, explain how loss to follow-up was addressed  *Case-control study*—If applicable, explain how matching of cases and controls was addressed  *Cross-sectional study*—If applicable, describe analytical methods taking account of sampling strategy | 14  17 | Methods – Statistical analyses (ll. 344-346)  Results – Attrition analyses |
|  |  | (*e*) Describe any sensitivity analyses | 15 | Methods – Statistical analyses (ll. 365-369) |
| Results | | | | |
| Participants | 13* | (a) Report numbers of individuals at each stage of study—eg numbers potentially eligible, examined for eligibility, confirmed eligible, included in the study, completing follow-up, and analysed | 11-12 | Methods – Sample, including Fig 2. Flowchart of retention rate and exclusion criteria resulting in final sample |
|  |  | (b) Give reasons for non-participation at each stage | 11-12 | Methods – Sample, including Fig 2. Flowchart of retention rate and exclusion criteria resulting in final sample |
|  |  | (c) Consider use of a flow diagram | 12 | Methods – Sample, Fig 2. Flowchart of retention rate and exclusion criteria resulting in final sample |
| Descriptive data | 14* | (a) Give characteristics of study participants (eg demographic, clinical, social) and information on exposures and potential confounders | 18-21 | Results – Descriptive statistics |
|  |  | (b) Indicate number of participants with missing data for each variable of interest | 20-21 | Results – Descriptive statistics: Table 1. Descriptive statistics; Table 2. Descriptive statistics for division of housework and childcare before and at the early stage of the COVID-19 pandemic |
|  |  | (c) *Cohort study*—Summarise follow-up time (eg, average and total amount) | 9-11 | Methods – Study design |
| Outcome data | 15* | *Cohort study*—Report numbers of outcome events or summary measures over time | 18-27 | Results |
|  |  | *Case-control study—*Report numbers in each exposure category, or summary measures of exposure |  |  |
|  |  | *Cross-sectional study—*Report numbers of outcome events or summary measures |  |  |
| Main results | 16 | (*a*) Give unadjusted estimates and, if applicable, confounder-adjusted estimates and their precision (eg, 95% confidence interval). Make clear which confounders were adjusted for and why they were included | 18-27  22  13-14 | Results, esp. Table 5. Prospective associations between predictors and linear change of the relationship satisfaction while adjusting for covariates.  Results – Multicollinearity; Table 2. Correlations between predictors and covariates for the final sample  Methods – Measures (ll. 321-328) |
|  |  | (*b*) Report category boundaries when continuous variables were categorized | 12-13 | Methods – Measures (ll. 294-320) |
|  |  | (*c*) If relevant, consider translating estimates of relative risk into absolute risk for a meaningful time period |  |  |
| Other analyses | 17 | Report other analyses done—eg analyses of subgroups and interactions, and sensitivity analyses | 22-27 | Results, esp. total sample vs. multi-group model and for sensitivity analyses) |
| Discussion | | | | |
| Key results | 18 | Summarise key results with reference to study objectives | 28 | Discussion – Summary of findings |
| Limitations | 19 | Discuss limitations of the study, taking into account sources of potential bias or imprecision. Discuss both direction and magnitude of any potential bias | 32-34 | Discussion – Strengths and limitations |
| Interpretation | 20 | Give a cautious overall interpretation of results considering objectives, limitations, multiplicity of analyses, results from similar studies, and other relevant evidence | 28-32 | Discussion |
| Generalisability | 21 | Discuss the generalisability (external validity) of the study results | 32-34 | Discussion (ll. 764-774) |
| Other information | |  | | |
| Funding | 22 | Give the source of funding and the role of the funders for the present study and, if applicable, for the original study on which the present article is based | Other information | The DREAM study is funded by the Deutsche Forschungsgemeinschaft (DFG, German Research Foundation) [grant numbers GA 2287/4-1 and GA 2287/4-2]. Susan Garthus-Niegel is a management committee member of COST action CA18211. The Article Processing Charges (APC) were funded by the joint publication funds of the TU Dresden, including Carl Gustav Carus Faculty of Medicine, and the SLUB Dresden as well as the Open Access Publication Funding of the DFG. The funders had no role in study design, data collection and analysis, decision to publish, or preparation of the manuscript. |

*Give information separately for cases and controls in case-control studies and, if applicable, for exposed and unexposed groups in cohort and cross-sectional studies.

**Note:** An Explanation and Elaboration article discusses each checklist item and gives methodological background and published examples of transparent reporting. The STROBE checklist is best used in conjunction with this article (freely available on the Web sites of PLoS Medicine at http://www.plosmedicine.org/, Annals of Internal Medicine at http://www.annals.org/, and Epidemiology at http://www.epidem.com/). Information on the STROBE Initiative is available at www.strobe-statement.org.
